# Recent progress on tyrosine kinase inhibitors resistance in renal cell carcinoma: another brick in the wall?

**DOI:** 10.20517/cdr.2025.157

**Published:** 2025-10-15

**Authors:** Zirui Dong, Shoukang Li, Mingfeng Li, Meiyin Fan, Kailei Chen, Anshu Li, Keshan Wang, Xiaoping Zhang

**Affiliations:** ^1^Department of Urology, Union Hospital, Tongji Medical College, Huazhong University of Science and Technology, Wuhan 430022, Hubei, China.; ^2^Department of Thoracic Surgery, Union Hospital, Tongji Medical College, Huazhong University of Science and Technology, Wuhan 430022, Hubei, China.; ^3^Health Management Center, Union Hospital, Tongji Medical College, Huazhong University of Science and Technology, Wuhan 430022, Hubei, China.; ^4^Department of Gastrointestinal Surgery, Union Hospital, Tongji Medical College, Huazhong University of Science and Technology, Wuhan 430022, Hubei, China.; ^5^Hubei Key Laboratory of Regenerative Medicine and Multi-disciplinary Translational Research (Huazhong University of Science and Technology), Wuhan 430022, Hubei, China.; ^#^These authors contributed equally to this work.

**Keywords:** Tyrosine kinase inhibitors (TKIs), renal cell carcinoma (RCC), drug resistance

## Abstract

Renal cell carcinoma (RCC), the predominant form of kidney cancer, accounts for 90% of cases and poses significant clinical challenges due to frequent late-stage or metastatic presentation. Based on literature and surveillance data from 2020 to 2025, despite therapeutic advancements, metastatic RCC still exhibits a dismal 5-year survival rate. While tyrosine kinase inhibitors (TKIs) targeting vascular endothelial growth factor/platelet-derived growth factor pathways have been a cornerstone of RCC treatment, their efficacy is limited by acquired resistance, necessitating novel strategies to improve patient outcomes. This review synthesizes advancements from 2020 to 2025 in understanding and overcoming TKI resistance in RCC. We explored emerging mechanisms of resistance, including tumor microenvironment remodeling, metabolic reprogramming, and activation of alternative survival pathways. Furthermore, we evaluated innovative therapeutic approaches. By consolidating recent insights, this review highlights promising strategies to circumvent resistance and underscores the importance of personalized, mechanism-driven therapies. Our analysis aims to inform future research directions and clinical translation, ultimately advancing the management of TKI-resistant RCC.

## INTRODUCTION

According to the American Cancer Society, an estimated 80,980 new cases and 14,510 deaths from kidney cancer were expected in the United States in 2025^[1]^. Worldwide, kidney cancer represented the 14th most frequently diagnosed malignancy, with more than 400,000 new cases in 2020^[2,3]^. Among all subtypes, renal cell carcinoma (RCC) is the predominant form, accounting for approximately 90% of cases^[4]^. RCC is often asymptomatic in its early stages^[5]^; however, approximately 30%-60% of patients present with metastatic disease at initial diagnosis according to different studies^[6,7]^. Moreover, almost 20% of patients with complete surgical removal of the primary tumor will develop metastatic disease^[8]^. The prognosis of metastatic RCC (mRCC) remains poor despite advancements in targeted therapy and immunotherapy, with a 5-year survival rate of approximately 17%^[9]^. The most common histological subtype of RCC, clear cell RCC (ccRCC), comprises about 70%-80% of cases and is characterized by frequent genetic alterations in the von Hippel-Lindau (VHL) tumor suppressor gene, leading to dysregulated hypoxia-inducible factor (HIF) signaling and angiogenesis^[10,11]^. These characteristics provide potential therapeutic targets for RCC.

The treatment landscape for advanced and mRCC has evolved significantly over the past decade, shifting from monotherapy with tyrosine kinase inhibitors (TKIs) to combination regimens incorporating immune checkpoint inhibitors (ICIs)^[12]^. Currently, the standard first-line therapies for advanced RCC primarily include TKI-based regimens, ICI-based combinations, or a combination of both^[13]^. TKIs, such as sunitinib, pazopanib, cabozantinib, lenvatinib, and axitinib, target vascular endothelial growth factor receptors (VEGFRs), platelet-derived growth factor receptors (PDGFRs), and other tyrosine kinases involved in angiogenesis and tumor progression^[14]^. While TKIs remain the mainstay of RCC treatment, their efficacy is inevitably limited by the development of acquired resistance after modest benefits in terms of disease-free progression^[15]^. As a result, TKI resistance remains a major challenge for the survival of RCC patients.

In this review, we provide a comprehensive update on the latest advancements in overcoming acquired resistance to TKIs in RCC over the past five years (2020-2025). We summarize recent insights into the underlying mechanisms driving resistance, including the evolving understanding of tumor microenvironmental adaptations, metabolic reprogramming, and alternative survival pathways. More importantly, we highlighted the diverse strategies researchers have explored in recent years to counteract TKI resistance, ranging from novel drug combinations to emerging therapeutic approaches that targeted previously unrecognized vulnerabilities in RCC. By consolidating these findings, we aimed to provide a valuable resource for guiding future research in the fight against TKI resistance in RCC.

## TYPES OF TKIS IN THE TREATMENT OF RCC AND THEIR MECHANISM

**Figure 1:**
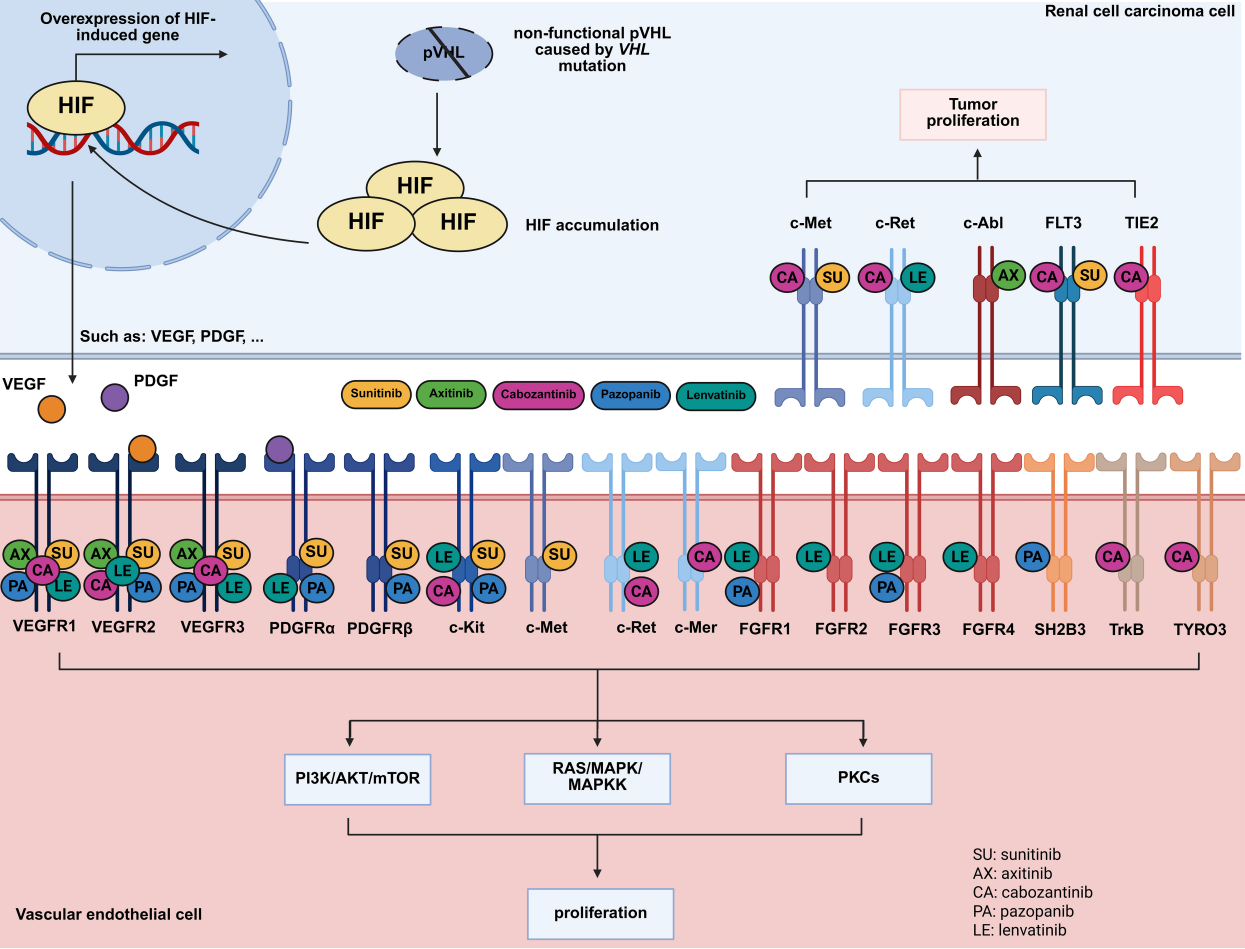
TKIs used in the treatment of RCC and their mechanisms. Created in BioRender. Dong, Z. (2025) https://BioRender.com/34g6akl. TKIs: Tyrosine kinase inhibitors; RCC: renal cell carcinoma.

Although different TKIs share common targets, their resistance mechanisms can vary significantly. Marona *et al.* (2022) demonstrated that while both sunitinib- and sorafenib-resistant RCC cells activated cellular-mesenchymal epithelial transition factor (c-MET) and interleukin-1 receptor-associated kinase 1 (IRAK1), their downstream effects diverged: sunitinib resistance led to E-cadherin expression, IL-6/IL-8 secretion, tumor senescence, and angiogenesis, whereas sorafenib resistance promoted mesenchymal traits, MMP9 secretion, and endothelial disruption, enhancing invasion^[16]^. These findings highlighted the heterogeneity of TKI resistance and suggested that mesenchymal epithelial transition factor (MET)-targeting agents such as cabozantinib could serve as late-line therapies to counteract bypass activation. Thus, the targets of different drugs should be outlined. Here, we summarized the mechanisms of action of all TKI agents recommended for the treatment of mRCC based on the latest clinical guidelines [Table 1 and Figure 1]^[12,13]^.

**Table 1:** TKIs used in the treatment of RCC and their targets (A: active; U: unknown)

**Drug name**	**Target(s)**	**Ref.**
Axitinib	VEGFR1(A), VEGFR2(A), VEGFR3(A), c-Abl(U)	[17,18]
Cabozantinib	VEGFR1(A), VEGFR2(A), VEGFR3(A), c-Kit(A), c-Met(A), c-Ret(A), TrkB(A), FLT3(A), TYRO3(A), c-Mer(A), TIE2(A)	[19,20]
Lenvatinib	VEGFR1(A), VEGFR2(A), VEGFR3(A), FGFR1(A), FGFR2(A), FGFR3(A), FGFR4(A), PDGFRα(A), c-Ret(A), c-Kit(A)	[21]
Pazopanib	VEGFR1(A), VEGFR2(A), VEGFR3(A), FGFR3(U), PDGFRα(A), PDGFRβ(A), c-Kit(A), FGFR1(U), SH2B3(U)	[22,23]
Sunitinib	VEGFR1(A), VEGFR2(A), VEGFR3(A), PDGFRα(A), PDGFRβ(A), FLT3(A), c-Met(U), c-Kit(A), CSF1R(A)	[18,24]

TKIs: Tyrosine kinase inhibitors; RCC: renal cell carcinoma; VEGFR: vascular endothelial growth factor receptor.

## THE MECHANISMS OF TKI RESISTANCE IN RCC: AN UPDATE (PRECLINICAL LEVEL: *IN VITRO* OR *IN VIVO*)

Many previous reviews have thoroughly summarized the mechanisms of TKI resistance in RCC, especially regarding sunitinib. Building on this foundation, we focused on studies from 2020-2025 that not only deepen our understanding of established resistance pathways but also introduce emerging mechanisms. Given the multifactorial nature of acquired resistance in RCC, some studies spanned multiple pathways; for clarity, we have categorized them according to their primary mechanism.

### Bypass activation

**Figure 2:**
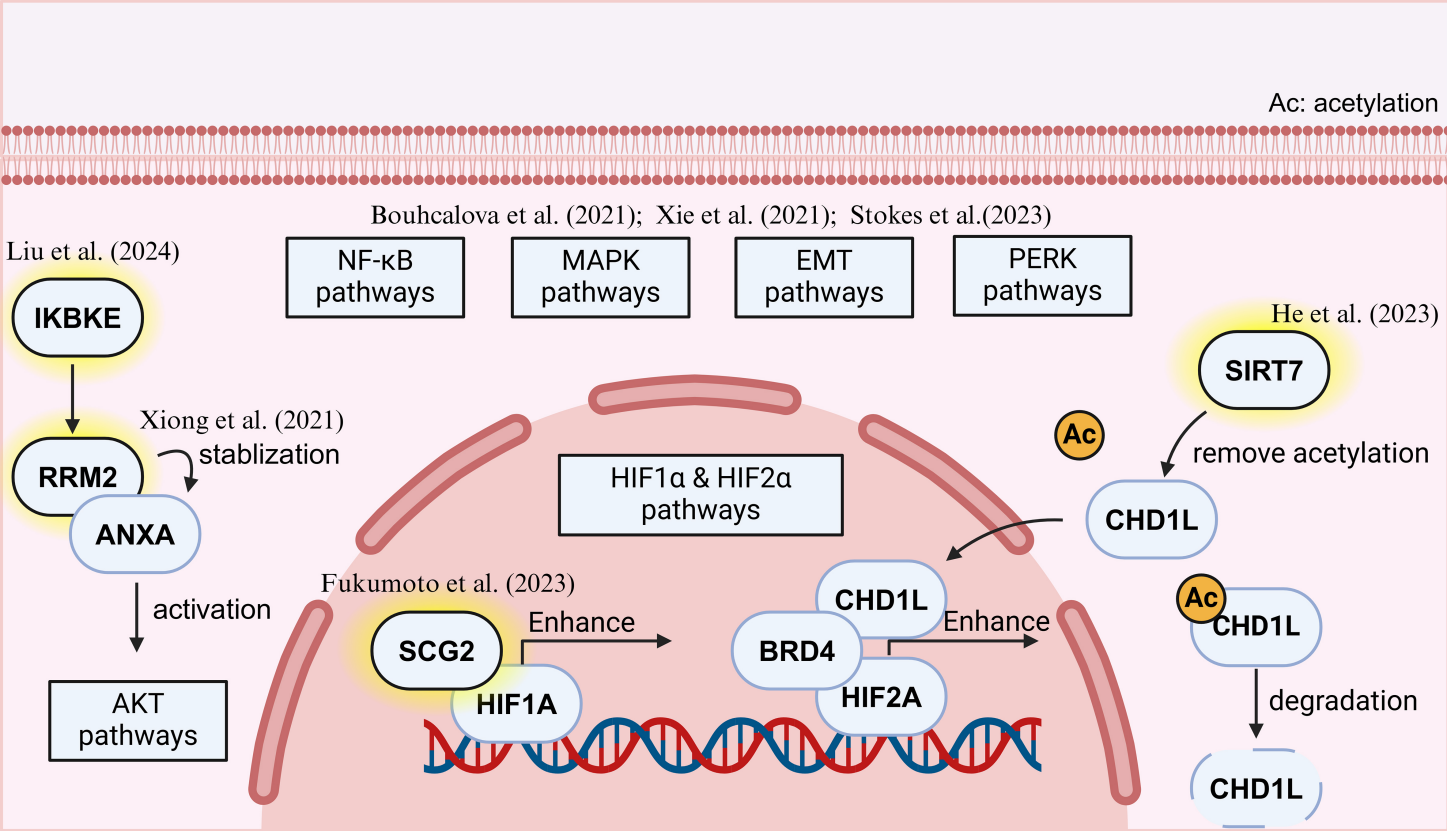
Recent progress of Bypass activation in TKI-resistant RCC. Created in BioRender. Dong, Z. (2025) https://BioRender.com/px2fkfx. TKI: Tyrosine kinase inhibitor; RCC: renal cell carcinoma.

Bypass activation is a crucial mechanism underlying acquired resistance to TKIs in RCC^[25]^. While TKIs primarily target VEGF/VEGFR signaling to inhibit tumor angiogenesis, prolonged drug exposure can lead to the activation of alternative pro-survival signaling cascades that allow tumor cells to evade VEGF inhibition. These compensatory pathways often involve receptor tyrosine kinases (RTKs) such as MET^[26]^, AXL^[27]^, and IGF-1R^[28]^, which can sustain tumor growth and angiogenesis independently of VEGF signaling. Understanding these bypass mechanisms is essential for developing rational combination therapies to overcome resistance and improve patient outcomes. Below, we summarize and compile studies from 2020-2025 on bypass activation leading to reduced sensitivity to TKIs in RCC [Figure 2].

Beyond MET signaling mentioned earlier^[16]^, other bypass pathways have been implicated in TKI resistance. Fukumoto *et al.* (2023) identified SCG2 as a novel mediator of sunitinib resistance, as it interacted with HIF-1α to activate the VHL/HIF/VEGF axis, thereby sustaining angiogenesis despite VEGFR inhibition^[29]^. Similarly, He *et al.* (2023) discovered that SIRT7 deacetylated CHD1L, stabilizing CHD1L protein levels by attenuating its ubiquitination levels. Accumulated CHD1L amplified HIF-2α signaling by interacting with HIF-2α, resulting in sunitinib resistance^[30]^.

The PI3K/AKT pathway has emerged as a central player in TKI resistance for a while^[31]^. New findings on the AKT pathway include: Xiong *et al.* (2021) reported that RRM2 stabilized ANXA1 to activate AKT signaling^[32]^, while Liu *et al.* (2024) further demonstrated that IKBKE phosphorylates RRM2, leading to sustained AKT activation in sunitinib-resistant (SR) RCC cells^[33]^. These findings underscore the significance of targeting PI3K/AKT signaling as a potential strategy to overcome TKI resistance.

Epithelial-to-mesenchymal transition (EMT) has also been identified as a crucial contributor to bypass activation^[34]^. Bouchalova *et al.* (2021) conducted proteomic analyses comparing TKI responders and non-responders, revealing that EMT was one of the most prominent pathways distinguishing resistant tumors^[35]^. This aligned with the observed increase in mesenchymal markers in sorafenib-resistant cells, further supporting the role of EMT in mediating escape from antiangiogenic therapy.

Beyond examining individual signaling pathways, a more comprehensive systems-level analysis was conducted by Xie *et al.* (2021), who established a SR cell-derived xenograft (CDX) model and identified enrichment of multiple resistance-associated pathways, including the aforementioned PI3K-AKT, HIF-1, NF-κB, and MAPK signaling^[36]^. The involvement of these pathways suggested a complex adaptive response in RCC that extended beyond VEGF signaling. Further supporting this notion, Stokes *et al.* (2023) demonstrated that treatment with multiple VEGFR-TKIs, including axitinib, cabozantinib, lenvatinib, and sunitinib, could activate PERK in 786-O RCC xenografts, implicating the unfolded protein response (UPR) as another potential mechanism of resistance^[37]^.

Together, these studies highlighted the intricate network of bypass mechanisms that sustained RCC progression despite VEGFR inhibition. The convergence of multiple signaling pathways - ranging from MET, PI3K/AKT to PERK and EMT - underscored the need for rational combination therapies targeting these alternative routes to effectively combat TKI resistance in RCC.

### RNAs (long non-coding RNA, circular RNA)

Circular RNAs (circRNAs) have emerged as key regulators of TKI resistance in RCC, influencing post-transcriptional gene regulation, metabolic reprogramming, and tumor microenvironment (TME) adaptation^[38]^. These non-coding RNAs function primarily by acting as molecular sponges for microRNAs (miRNAs) or interacting with RNA-binding proteins (RBPs), ultimately modulating critical signaling pathways that drive drug resistance^[39]^.

One of the interesting mechanistic insights into circRNA-mediated TKI resistance came from Huang *et al.* (2021), who identified circSNX6 as a molecular sponge for miR-1184. By sequestering miR-1184, circSNX6 alleviated its suppressive effect on GPCPD1, leading to an increase in intracellular lysophosphatidic acid (LPA) levels and enhanced sunitinib resistance^[40]^. In 2023, our study revealed that circPTPN12 promoted sunitinib resistance through the hnRNPM/IL-6/STAT3 signaling axis. Specifically, circPTPN12 enhanced hnRNPM binding to IL-6 mRNA, increasing its stability and sustaining STAT3 pathway activation, thereby highlighting the contribution of inflammatory cytokine signaling to TKI resistance^[41]^. In a 2023 study from our lab, circRARS was shown to bind the KH1-KH2 domains of IGF2BP3, enhancing its recognition of m6A-modified transcripts. This interaction promoted lipid accumulation in RCC cells and contributed to sunitinib resistance through downstream targets, underscoring the role of metabolic adaptation in therapeutic evasion^[42]^.

Long non-coding RNAs (lncRNAs) have recently been identified as critical regulators in the development of resistance to TKIs in RCC^[43]^. These lncRNAs modulate various cellular processes such as autophagy, protein stability, and oxidative stress, contributing to TKI resistance by interacting with RBPs, key transcription factors, and cellular pathways^[44]^.

In 2023, Pan *et al.* uncovered that IGFL2-AS1, a lncRNA, enhanced the expression of TP53INP2, which promoted autophagy, leading to sunitinib resistance^[45]^. In a separate study conducted by our lab in 2020, we identified lncRNA SNHG12 as a key player in sunitinib resistance. SNHG12 binds to SP1, preventing its ubiquitylation-dependent degradation. This stabilization of SP1 led to increased expression of CDCA3, a gene that played a key role in the cell cycle, and promoted resistance to sunitinib. The interaction between SNHG12 and SP1 underlined the importance of transcription factor regulation in mediating resistance in RCC^[46]^. Further investigation into the roles of lncRNAs in RCC TKI resistance revealed that lncRNA SNHG1 binds to PTBP1 and regulates the ATG7 axis, a crucial player in autophagy. This interaction not only enhanced autophagic flux but also contributed to RCC resistance to sunitinib by sustaining cellular survival under therapeutic pressure, as identified by Tian *et al.* in 2024^[47]^. In addition to autophagy, oxidative stress and ferroptosis have also been shown to be modulated by lncRNAs in RCC^[43,44]^. Pan *et al.* (2025) discovered that STX17-DT, a lncRNA upregulated in axitinib-resistant RCC cells, interacted with hnRNPA1, stabilizing IFI6 mRNA. This interaction led to increased ROS levels and suppression of ferroptosis, further contributing to drug resistance^[48]^. The study suggested that lncRNA-mediated modulation of oxidative stress pathways might represent a therapeutic target to overcome TKI resistance. Moreover, He *et al.* (2022) discovered that sunitinib could increase the expression of lncRNA-ECVSR, enhancing ERβmRNA and transcriptionally upregulating HIF-2α^[49]^.

**Figure 3:**
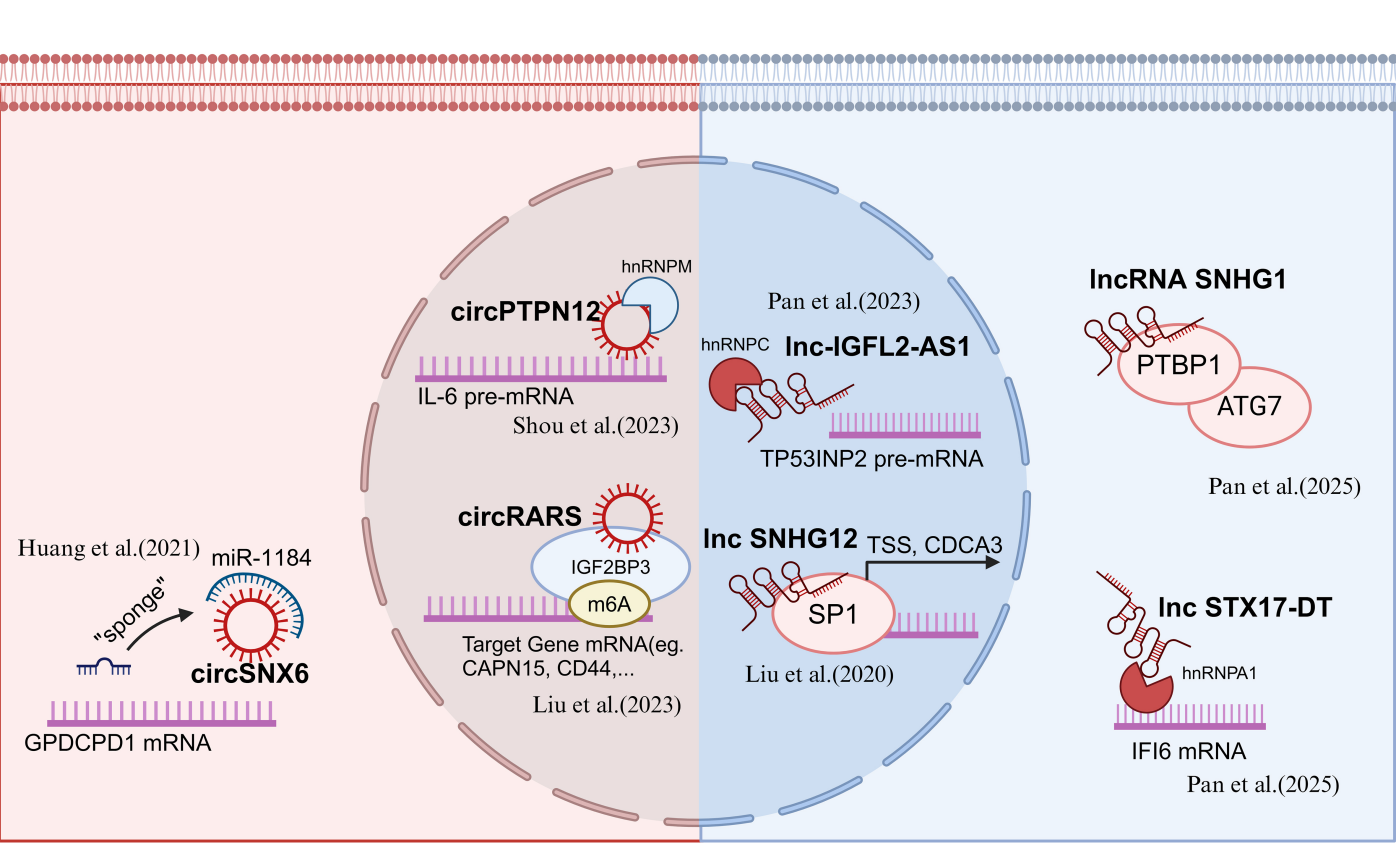
Recent updates on RNAs’ function in RCC TKI resistance. Created in BioRender. Dong, Z. (2025) https://BioRender.com/qmr14qn. RCC: Renal cell carcinoma; TKI: tyrosine kinase inhibitor.

These studies collectively emphasized the growing importance of circRNAs and lncRNAs as pivotal regulators of TKI resistance in RCC. By influencing key cellular processes, circRNAs and lncRNAs represented promising targets for novel therapeutic strategies aimed at overcoming resistance and improving the efficacy of TKI therapies in RCC [Figure 3].

### Lysosome

Lysosomal pumping/sequestration has recently emerged as a key mechanism contributing to resistance against TKIs in RCC^[50]^. This process involves the enhanced activity of lysosomes, which actively pump therapeutic agents out of the cytoplasm, thereby reducing drug efficacy.

In 2021, Li *et al.* demonstrated that long-term exposure to sunitinib induced lysosomal biosynthesis and exocytosis, thereby promoting sunitinib resistance. Under sunitinib treatment, TFE3, a transcription factor, continuously translocated into the nucleus, where it drove the expression of E-Syt1, an endoplasmic reticulum (ER) protein. E-Syt1 induced fragmentation of the ER, which in turn promoted lysosomal exocytosis. This process facilitated the active export of sunitinib from the cytoplasm, effectively lowering its intracellular concentration and reducing its therapeutic effects on RCC cells^[51]^. This study underscored the significant role of lysosomal pumping in mediating resistance to sunitinib in RCC, highlighting the importance of lysosomal dynamics in the development of drug resistance.

### Cell death (apoptosis, ferroptosis, cuproptosis, *etc.*)

Recent research has highlighted the critical role of various cell death pathways, including ferroptosis, apoptosis, and cuproptosis, in the development of resistance to TKIs in RCC.

Chen *et al.* (2024) integrated single-cell RNA sequencing (scRNA-seq) data from both pre-treatment and post-treatment surgical samples with SR ccRCC cell lines. They found that ferroptosis, a form of regulated cell death characterized by iron-dependent lipid peroxidation, played a key role in mediating resistance to sunitinib. Specifically, the inflammatory cytokine IL-6 was shown to reverse ferroptosis, thereby promoting RCC resistance to sunitinib^[52]^. This suggested that targeting ferroptosis could be a promising strategy to overcome TKI resistance in RCC. Additionally, our lab (2023) discovered that AIM2, a pattern recognition receptor, promoted sunitinib resistance by enhancing the phosphorylation and proteasomal degradation of FOXO3a, a transcription factor involved in ferroptosis. This process inhibited the transcriptional activation of ACSL4, a key regulator of ferroptosis, thereby reducing ferroptosis and conferring resistance to sunitinib in RCC cells^[53]^. In addition, the lncRNA STX17-DT mediates sunitinib resistance in RCC by regulating ferroptosis, highlighting its role in cell death−related mechanisms of TKI resistance^[48]^. Furthermore, Wu *et al.* (2023), through multi-omics analysis, identified PDHB, a critical gene involved in cuproptosis (copper-induced cell death), as a key factor in overcoming sunitinib resistance in RCC. Activation of PDHB promoted copper-induced cell death, providing insight into targeting cell death pathways to overcome TKI resistance^[54]^. Moreover, Zeng *et al.* (2024) identified the regulatory role of O-GlcNAcylation on RIPK1, a key protein in cell death regulation. The modification of RIPK1 by O-GlcNAcylation influenced the assembly of the RIPK1/FADD/Caspase8 complex and activated the NF-κB pathway, suppressing RIPK1-dependent apoptosis induced by sunitinib treatment^[55]^. This mechanism may contribute to the resistance of RCC cells to sunitinib by preventing apoptosis, a form of programmed cell death.

### Metabolic reprogramming

**Figure 4:**
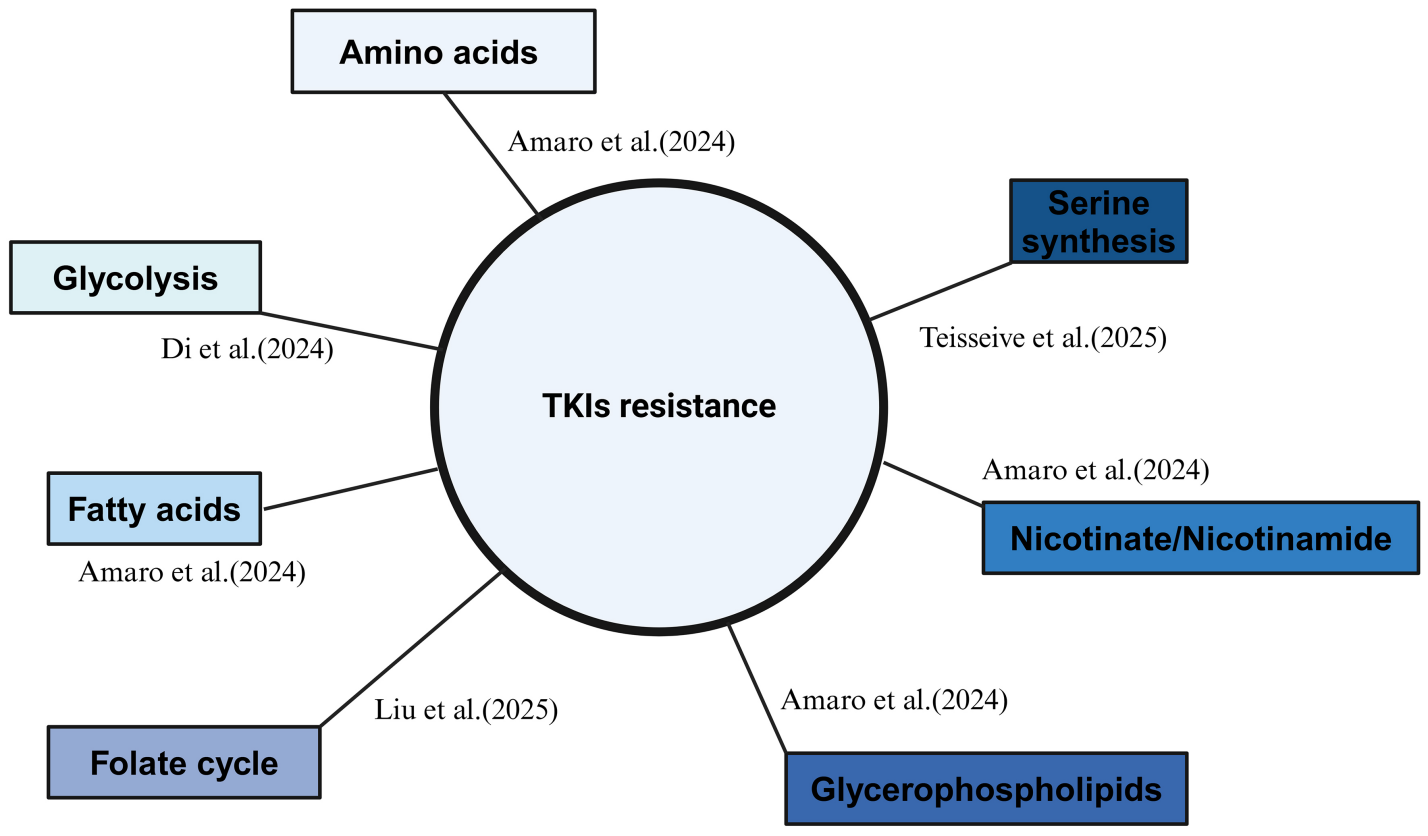
Recent updates on metabolic shifting during RCC TKI resistance. Created in BioRender. Dong, Z. (2025) https://BioRender.com/vs7bn3w. RCC: Renal cell carcinoma; TKI: tyrosine kinase inhibitor.

Recent studies have increasingly highlighted metabolic reprogramming as a crucial factor in the development of resistance to TKIs in RCC. Changes in metabolic pathways, such as glycolysis, amino acid metabolism, and fatty acid metabolism, have been shown to play pivotal roles in mediating resistance mechanisms in RCC^[56]^ [Figure 4].

Di *et al.* (2024) performed scRNA-seq on 14 RCC patients and identified glycolysis as a central metabolic pathway in the development of TKI resistance in RCC. This was mediated through the activation of the AKT/mTOR/HIF-1α pathway, emphasizing the metabolic shift that RCC cells underwent during TKI resistance^[57]^. The study underlined how metabolic alterations contribute to the survival and proliferation of RCC cells in the presence of TKIs. In another systematic study, Amaro *et al.* (2024) explored the metabolic profiling of sunitinib- and pazopanib-resistant RCC cells, analyzing both intracellular and extracellular metabolomes^[58]^. While the study’s limitation lies in its *in vitro* approach, it revealed several metabolic pathways related to TKI resistance in RCC. These pathways included those involving amino acids, glycerophospholipids, fatty acids, and nicotinate/nicotinamide. Interestingly, the metabolic changes in fatty acid metabolism observed in this study aligned with findings from our lab^[59]^, suggesting a common metabolic shift in TKI-resistant RCC. Moreover, these RNA-related studies further underscore the critical role of lipid metabolism in mediating TKI resistance in RCC^[40,42]^.

In another study, Liu *et al.* (2025) identified MTHFD2 as a key player in the metabolic adaptation of RCC cells to sunitinib resistance. MTHFD2 drove the folate cycle, stimulating the upregulation of UDP-GlcNAc and promoting c-Myc O-GlcNAcylation, both of which contributed to resistance to sunitinib^[60]^. This mechanism highlighted the role of epigenetic regulation through metabolic intermediates in modulating TKI resistance.

Additionally, Teisseire *et al.* (2025) observed that sunitinib treatment induced a metabolic shift in RCC cells, leading to increased serine synthesis. The GCN2-eIF2α-ATF4 stress response pathway was identified as the key link between sunitinib treatment and elevated serine production, promoting nucleotide synthesis and supporting the metabolic needs of resistant RCC cells^[61]^.

These findings underscore the critical role of metabolic reprogramming in RCC resistance to TKIs. The ability of RCC cells to adapt their metabolism in response to treatment not only enabled their survival but also enhanced their resistance to TKIs. Targeting these metabolic pathways may provide promising therapeutic strategies to overcome resistance in RCC patients treated with TKIs.

### Angiogenic switch

The development of TKI resistance in RCC is often accompanied by an angiogenic switch, where tumors adapt to prolonged antiangiogenic therapy by modifying their vascular microenvironment^[62]^. Several recent studies have elucidated key mechanisms through which RCC cells reprogram angiogenesis to evade TKI treatment.

Xuan *et al.* (2021) reported that exosomal miR-549a was significantly reduced in TKI-resistant RCC, promoting angiogenesis and vascular permeability by upregulating HIF-1α in endothelial cells, thus contributing to resistance against antiangiogenic therapies^[63]^. The previously mentioned study has also shown that lncRNA-ECVSR modulates RCC sensitivity to sunitinib via transcriptional regulation of HIF-2α, a key signaling pathway governing angiogenesis^[49]^.

Similarly, Gu *et al.* (2020) demonstrated that estrogen receptor beta (ERβ) transcriptionally upregulates ANGPT-2, leading to Tie-2 phosphorylation, a key event in promoting angiogenesis and reinforcing resistance to sunitinib treatment^[64]^. This study highlighted the intricate role of hormonal signaling in modulating angiogenic pathways and TKI resistance.

Moreover, in a clinical study, Zhao *et al.* (2021) analyzed tumor samples from sunitinib-responsive and non-responsive patients and identified CTCF as a crucial factor in promoting sunitinib resistance by enhancing angiogenesis^[65]^. This finding underscored the significance of epigenetic regulators in driving vascular adaptation in RCC under TKI therapy.

These studies collectively indicated that RCC cells undergoing TKI resistance reactivated pro-angiogenic pathways to sustain tumor growth despite antiangiogenic treatment. Targeting these alternative angiogenic mechanisms may offer new therapeutic strategies to counteract TKI resistance in RCC.

### Epigenetic modifying

Epigenetic modifications play a crucial role in the development of TKI resistance in RCC by regulating gene expression without altering the DNA sequence. In 2022, Chen *et al.* found that TRAF1 is upregulated in SR cells and clinical samples due to elevated N6-methyladenosine (m6A) modification in a METTL14-dependent manner, underscoring the role of RNA methylation in TKI resistance^[66]^. In 2020, our lab discovered that methylation of the PCK2 promoter leads to ER stress in RCC cells, ultimately contributing to sunitinib resistance^[67]^. More recently, in 2024, by establishing an *in vivo* sunitinib resistance model, our lab identified that MIER2 facilitates p53 deacetylation by interacting with HDAC1, leading to resistance in RCC^[68]^.

Together, these studies revealed that epigenetic regulation, including m6A RNA modification, DNA methylation, and histone deacetylation, constituted a key mechanism underlying TKI resistance in RCC, offering potential therapeutic targets for overcoming resistance.

### Drug resistance transmission

The transmission of drug sensitivity was proposed as a concept in microbiome; however, studies have found that drug resistance could be transmitted between cancer cells and has been validated in other studies^[69,70]^. In recent years, a few studies have reported that the sensitivity of RCC to TKIs can be transmitted through extracellular vesicles (EVs)^[27,71]^. Notably, a 2021 study demonstrated that treatment with sunitinib and axitinib led to increased EV secretion in RCC cells, along with metabolic reprogramming of these vesicles, particularly toward glycolysis^[72]^. This finding strongly suggested the potential involvement of EVs in RCC’s adaptive response and the transmission of resistance to TKIs.

For instance, as discussed earlier, Pan *et al.* (2025) discovered that STX17-DT, beyond its intrinsic role in TKI resistance within RCC cells, could be packaged into EVs via hnRNPA1, enabling the transmission of axitinib resistance to other cells^[48]^. Similarly, in a 2023 study mentioned above, Pan *et al.* demonstrated that IGFL2-AS1, despite its intracellular function previously mentioned in this review, can also be encapsulated into EVs through hnRNPC, thereby facilitating the transfer of sunitinib resistance between RCC cells^[45]^.

### Cancer stem cell phenotype

Cancer stem-like cells (CSCs) possess self-renewal capabilities, plasticity, and the ability to survive under therapeutic stress, contributing to tumor recurrence and drug resistance^[73,74]^. In 2022, Guo *et al.* found that under hypoxic conditions, the androgen receptor regulated the stem cell phenotype of RCC through the lncTCFL5-2/YBX1/SOX2 signaling axis, thereby enhancing RCC cell resistance to sunitinib^[75]^. Similarly, as discussed above, sunitinib-triggered activation of lncRNA-ECVSR/ERβ/HIF-2α signaling could result in an enhanced cancer stem cell phenotype, which ultimately leads to resistance^[49]^.

### TME

**Figure 5:**
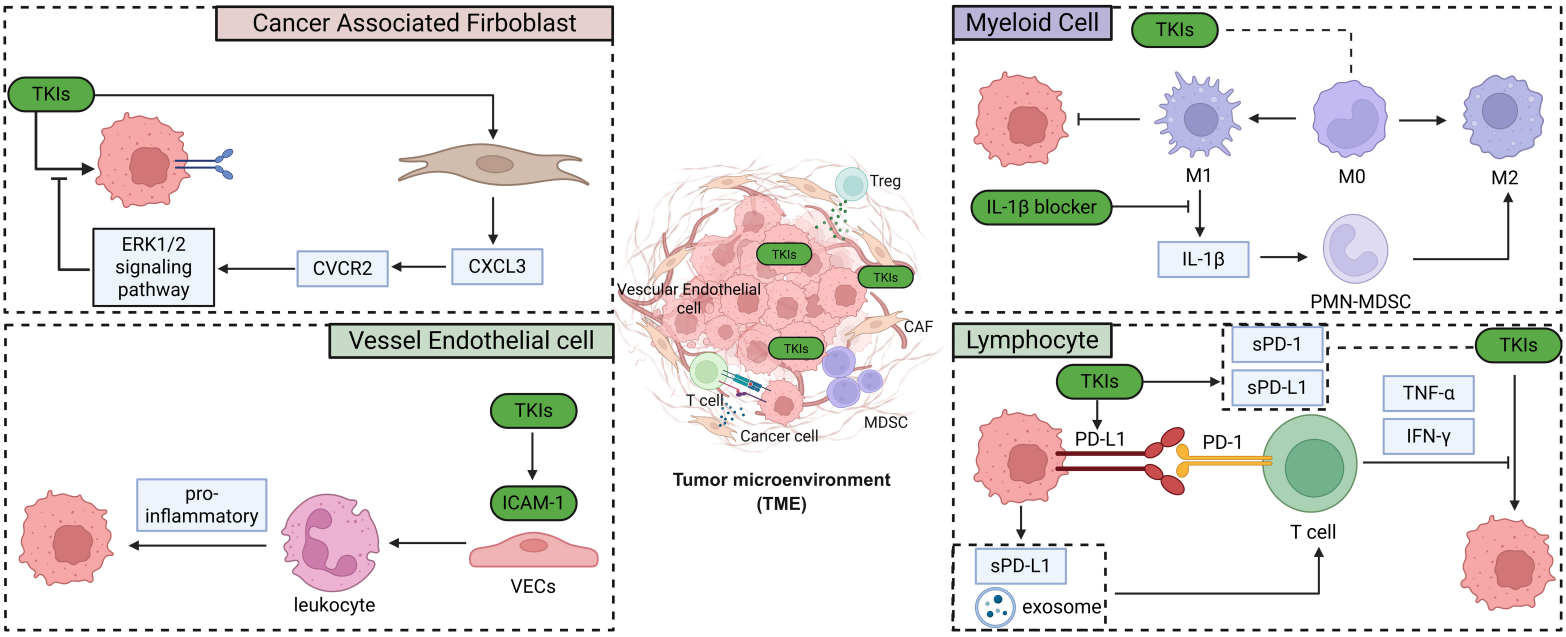
Recent updates on the relationship of TME with RCC TKI resistance. Created in BioRender. Dong, Z. (2025) https://BioRender.com/kqpg9zr. TME: Tumor microenvironment; RCC: renal cell carcinoma; TKI: tyrosine kinase inhibitor.

As another malignancy treated with the combination of ICIs and TKIs, lung cancer has been extensively investigated, with numerous studies reporting that alterations in the TME profoundly modulate tumor responses to TKIs^[76,77]^. Recent studies have also highlighted that changes in TME components play a pivotal role in shaping drug responsiveness in RCC [Figure 5]. However, a greater number of influential studies on this topic were conducted earlier than 2020^[78-81]^, whereas recent research has shown a decline in investigations of the interactions between TKIs and the TME^[36,82]^. In the following sections, we will discuss recent progress on how individual components of the TME influence the sensitivity of RCC to TKI therapy.

### Myeloid cell

As the predominant component of the TME, myeloid cells are widely recognized to be closely associated with the response of RCC to TKI therapy^[83]^. Recent studies have shown that M0 macrophages within the TME are closely associated with tumor responsiveness to both immunotherapy and TKI treatment^[84]^. Aggen *et al.* demonstrated that IL-1β blockade reduced polymorphonuclear MDSC (PMN-MDSC) infiltration and, in combination with cabozantinib, enhanced antitumor efficacy. These effects were accompanied by decreased immunosuppressive MDSCs and increased M1-like TAMs within the TME. Collectively, these findings provide the first evidence of potential synergy between IL-1β inhibition and VEGF-targeted TKI therapy, although the precise mechanisms - particularly how MDSC reduction and M1-like TAM enrichment contribute to this synergy - remain to be elucidated^[85]^.

### Lymphocyte

Recent studies have also shown that TKI treatment in RCC can induce PD-L1 expression and the release of soluble PD-L1 from tumor cells, thereby impairing T cell activation, reducing cytokine production, and decreasing the proportion of activated T cells^[86]^. Specifically, Liu *et al.* demonstrated that in SR RCC, NFAT1 is stabilized via PI3K/AKT/GSK-3β signaling and FOXA1/SETD2-mediated downregulation of FBW7, leading to increased PD-L1 expression. This mechanism promotes immune evasion by impairing T cell activity, underscoring NFAT1 as a key link between TKI resistance and T cell suppression^[87]^. Similarly, Greenberg *et al.* discovered that exosomes from SR RCC cells impair T cell function, exhibiting cytotoxicity that correlates with elevated PD-L1 levels compared with sunitinib-sensitive (SS) exosomes^[88]^. Interestingly, the expression levels of soluble PD-L1 and soluble PD-1 have also been identified as independent prognostic factors for progression-free survival (PFS) in mRCC patients treated with sunitinib^[89]^. Moreover, studies also identified several hub genes that simultaneously regulate the responsiveness of RCC cells to both TKIs and ICIs, suggesting that tumor cells may employ convergent adaptive mechanisms in response to therapy-induced alterations within the microenvironment^[32,54,90-92]^. However, it is noteworthy that the regulatory trends of certain hub genes in RCC are not always concordant between these two therapeutic modalities. For instance, in 2025, Yang *et al.* reported that high expression of COL6A2 was associated with increased sensitivity to sunitinib but reduced responsiveness to ICIs, underscoring the complexity of the underlying mechanisms^[93]^. In addition to tumor cell–intrinsic alterations influencing lymphocyte function and therapeutic responsiveness, the nature of T cell infiltration has also been linked to TKI efficacy in RCC. A clinical multi-omics study in 2020 demonstrated that infiltration of CD39^+^CD8^+^ T cells was associated with poorer responses to TKI therapy, potentially due to reduced levels of TNF-α and IFN-γ^[94]^. This is particularly interesting, as another independent study demonstrated that TKI treatment alters the heterogeneity of T cell infiltration in RCC, especially by affecting the ratio of CD39^+^CD8^+^ T cells^[95]^.

### Vascular endothelial cells

As the principal targets of TKIs in RCC, vascular endothelial cells (VECs) play a pivotal role in shaping the TME, and endothelial cell responsiveness to TKIs represents a key mechanism underlying both TKI resistance and immunotherapy responsiveness^[96]^. In 2023, researchers discovered that treatment with VEGF-targeted agents such as sunitinib or bevacizumab restored ICAM-1 expression in endothelial cells, promoted leukocyte adhesion and infiltration, and generated a pro-inflammatory tumor milieu. These findings highlight the potential of combining antiangiogenic agents with immunotherapy to enhance antitumor immune responses^[97]^.

### Cancer-associated fibroblasts

Activated fibroblasts educated by cancer cells, known as cancer-associated fibroblasts (CAFs), exhibit sustained activation properties^[98]^. In RCC, CAFs have been reported to display pronounced heterogeneity and to contribute to tumor progression by promoting proliferation^[99]^, enhancing stemness^[100]^, suppressing immune cell functions^[101]^, and - most relevant to this review - mediating resistance to TKI therapy^[102]^. In 2024, Wang *et al.* discovered that CAFs could secrete CXCL3 and activate its receptor CXCR2 on RCC, resulting in the activation of the downstream ERK1/2 signaling pathway, thus promoting RCC sunitinib resistance^[103]^.

The infiltration of CAFs is not merely a unidirectional process leading to RCC resistance to TKI therapy^[104]^. A 2021 study analyzing three cohorts demonstrated that VEGFR-TKI treatment increases CAF infiltration within the TME, thereby mediating resistance. This finding highlights a more complex interplay between TKI therapy and CAFs^[105]^.

Above all, a comprehensive study by Stupichev *et al.* developed an AI-driven multimodal algorithm based on transcriptomic data from 14 cohorts, which constructed a harmonized immune tumor microenvironment (HiTME) landscape to predict RCC responsiveness to both ICI and TKI therapies^[106]^.

## RECENT REPORTS ON OVERCOMING TKI RESISTANCE IN RCC (PRECLINICAL LEVEL: *IN VITRO* OR *IN VIVO*)

From a translational perspective, recent research has increasingly focused on strategies to overcome TKI resistance in RCC. Building on the summarized mechanisms, the following section reviews key therapeutic advances proposed from 2020 to 2025, with an emphasis on their clinical relevance. These strategies are summarized in Table 2.

**Table 2:** Recent strategies proposed by researchers to overcome TKI resistance in RCC

	**Mechanism**	**Ref.**
**Natural products**		
ATL-I	Degradation of EPAS1	[107]
Wogonin	Inhibits CDK4-RB	[108]
PTE, 6-S	Inhibit Ras/ERK and Akt/mTOR	[109]
**Small molecules**		
**Kinase inhibitors**		
**Targeting PI3K**		
STS	Inhibits PI3K/Akt and MDR1	[110]
TGX-221	Inhibits PI3Kβ	[111]
Pictilisib	Pan-PI3K inhibitor	[112]
CYT387	IKBKE inhibitor	[33]
**Targeting mTOR**		
sapanisertib	Inhibits mTORC1/2	[113]
Rapalink-1	Inhibits mTOR, MAPK, ErbB, and ABC transporters	[114]
**Targeting ERK**		
Calcium saccharate	Enhances DUSP6, intervening in ERK/AKT	[115]
**Targeting drug-resistant mutations**		
15e	Targets MET V1238I and Y1248H	[111]
**Other kinase inhibitors**		
Nilotinib	Degradation of MCL-1	[116]
AZD4547	FGFR inhibitor	
**Nuclear receptor-targeting inhibitors**		
PHTPP	ERβ antagonist	[49]
Faslodex		[64]
XCT-790	Inhibits ERRα	[117]
**HSP and stress response inhibitors**		
AUY922	Targets HSP90	[118]
HC-5404	Inhibits PERK	[37]
**Metabolic and angiogenesis pathway inhibitors**		
Sitagliptin	Inhibits DPP4	[119]
Disulfiram	Inhibits ALDH1A	[119]
Simvastatin	Inhibits SREBP-1	[120]
Angiotensin-(1-7)		[121]
Meletin	HOOK1 agonist	[122]
CHD1Li	Targets CHD1L, influences HIF-2α	[30]
EMφ-siMTHFD2-MnO2@Suni	Targets folate-nucleotide metabolism	[123]
**Drug repurposing**		
Penfluridol	Induces apoptosis	[124]
KTZ	Inhibits ERK1/2	[125]
**RNA interference**		
ASOs	Silences IGFL2-AS1	[45]
St/siVEGFR-2@PCN-224@HA	RNAi + photodynamic	[126]
**Others**		
[177Lu]Lu-cG250 RIT		[127]
Lm-LLO-CD105A	Targets CD105	[128]

TKI: Tyrosine kinase inhibitor; RCC: renal cell carcinoma; ATL-I: Atractylenolide I; EPAS1: endothelial PAS domain protein 1, also known as hypoxia-inducible factor-2α, HIF-2α; CDK4-RB: cyclin-dependent kinase 4–retinoblastoma protein axis; PTE: Pterostilbene; 6-S: 6-Shogaol; ERK: extracellular signal-regulated kinase; Akt: protein kinase B; mTOR: mechanistic target of rapamycin; PI3K: phosphoinositide 3-kinase; STS: staurosporine; MDR1: ATP-dependent translocase ABCB1; IKBKE: inhibitor of nuclear factor kappa-B kinase subunit epsilon; MAPK: mitogen-activated protein kinase; ErbB: erythroblastic leukemia viral oncogene homolog; ABC: ATP-binding cassette; MET: mesenchymal-epithelial transition factor/hepatocyte growth factor receptor; MCL-1: myeloid cell leukemia-1; FGFR: fibroblast growth factor receptor; PHTPP: 4-[2-phenyl-5,7-bis(trifluoromethyl)pyrazolo[1,5-a]pyrimidin-3-yl]phenol; ERβ: estrogen receptor beta; ERRα: estrogen-related receptor alpha; PERK: Eukaryotic translation initiation factor 2-alpha kinase 3; HSP: heat shock protein; DPP4: dipeptidyl peptidase IV; ALDH1A: aldehyde dehydrogenase 1 family member A; SREBP-1: sterol regulatory element-binding protein 1; HIF: hypoxia-inducible factor; KTZ: ketoconazole; ASOs: antisense oligonucleotides.

### Natural products

Natural products offer unique chemical diversity, high biological activity, and better target selectivity, making them valuable in drug discovery. Their favorable pharmacokinetics and safety profiles enhance their potential for developing novel therapeutics^[129]^.

Several natural compounds have shown potential in reversing TKI resistance in RCC by targeting key oncogenic pathways. Recently, Atractylenolide I (ATL-I), derived from Atractylodes macrocephala, was found to reverse sunitinib resistance by inhibiting EPAS1/HIF-2α-mediated VEGFA production and promoting the autophagic degradation of EPAS1 via lysosomal activation^[107]^. Similarly, Wogonin, an active component from Scutellaria baicalensis, restored sunitinib sensitivity by inhibiting the CDK4-RB pathway and inducing apoptosis^[108]^. Likewise, Honokiol, a phenolic compound, was identified as a potent inhibitor of c-Met-induced signaling, providing a potential strategy to overcome cabozantinib resistance^[130]^. More recently, Pterostilbene (PTE) and 6-Shogaol (6-S), natural phytochemicals found in edible sources, were shown to counteract sunitinib resistance by suppressing RLIP76-initiated Ras/ERK and Akt/mTOR pathways^[109]^.

### Small molecule inhibitor

#### Kinase inhibitors

Targeting PI3K In 2020, Kinoh *et al.* discovered that staurosporine (STS), a competitive ATP mimetic with broad kinase inhibition affinity, could overcome sunitinib resistance by inhibiting PI3K/Akt and MDR1. In addition, to overcome the severe off-targeting toxicities *in vivo*, they developed strategies for tumor-selective delivery by loading STS with polymeric micelles^[110]^. In the same year, Azad *et al.* discovered that the inhibition of phosphoinositide 3-kinase (PI3K)β, an isoform uniquely coupled to both RTK and GPCRs, combined with sunitinib could reduce microvessel turnover and decrease heterogeneity of the TME^[111]^. Moreover, using the validated Therapeutically Guided Multidrug Optimization (TGMO) method, Pictilisib (pan-phosphatidylinositol 3-kinase inhibitor) was selected as a promising low-dose drug for combination therapy, effective in both naïve and resistant tumors^[112]^.

Targeting mTOR Recently, Wu *et al.* tested ten drugs and selected combinations across six RCC PDX models, finding cabozantinib and mTORC1/2 sapanisertib to be the most effective combinational therapies in suppressing tumors from patients who had failed prior TKI and ICI treatments, by blocking the ERK pathway^[113]^. Similarly, in 2020, Kuroshima *et al.* tested Rapalink-1, a new generation of mTOR inhibitor, in the treatment of SR RCC. The results showed that rapalink-1 had greater effects than the current second-line treatment, temsirolimus, by not only suppressing the mTOR signaling pathway but also the MAPK signaling pathway, ErbB signaling pathway, and several drug-resistance-associated ABC transporters^[114]^. These findings provided valuable insights that the mTORC1/2 double inhibitor may be effective against TKI resistance, as the mTOR inhibitor is currently the second-line treatment of mRCC following TKI monotherapy or TKI + ICI treatment.

Targeting ERK In 2024, Liu *et al.* discovered that calcium saccharate could overcome sunitinib resistance by enhancing the expression of DUSP6, thus intervening in the ERK-AKT pathway^[115]^.

Targeting drug-resistant mutation In 2021, Li *et al.* synthesized a novel derivative of APG compound 15e, which demonstrated satisfying anti-proliferation effectivity against proven cabozantinib-resistant mutant MET V1238I and Y1248H^[131]^.

Other kinase inhibitors Liu *et al.* screened synergistic reagents of sunitinib from a compound library containing 1,374 FDA-approved drugs by *in vitro* cell viability evaluation. As a result, nilotinib stood out as a potential synergistic killer, rendering MCL-1 degradation and RCC autophagy to overcome sunitinib resistance^[116]^.

Another inhibitor screened as a promising drug to overcome sunitinib resistance in RCC is AZD4547, an FGFR inhibitor^[112]^.

#### Nuclear receptor-targeting inhibitors

Emerging evidence suggests that nuclear receptors play crucial roles in sunitinib resistance in RCC. As aforementioned, He *et al.* identified a positive-feedback loop between ERβ and lncRNA-ECVSR, where sunitinib-induced ERβ overexpression further upregulated lncRNA-ECVSR, promoting vasculogenic mimicry (VM). Disrupting this loop with the ERβ antagonist PHTPP enhanced sunitinib sensitivity and reduced VM formation^[49]^. Similarly, targeting the ERβ/ANGPT-2/Tie-2P signaling pathway with the FDA-approved anti-estrogen drug Faslodex has been proposed as a potential combination therapy to overcome sunitinib resistance^[64]^. Beyond ERβ, Feng *et al.* recently demonstrated that inhibiting ERRα acetylation-mediated autophagy-lysosome pathways by XCT-790 sensitized RCC cells to sunitinib, further supporting the therapeutic potential of nuclear receptor modulation in overcoming drug resistance^[117]^.

#### Heat shock protein and stress response inhibitors

Recently, Saito *et al.* discovered that heat shock transcription factor 4 (HSF4) is elevated in SR RCC cells and the combination of pazopanib with HSF4 knockdown reduced cell proliferation in SR cells^[90]^. Similarly, another inhibitor targeting heat shock protein HSP90, AUY922, demonstrated the ability to increase the sensitivity of ccRCC cells by targeting the HIF-1α/VEGFA/VEGFR pathway^[118]^.

As discussed above, to encounter PERK activation in TKIs-treated RCC, Stokes *et al.* discovered that PERK inhibitor HC-5404 enhanced the antiangiogenic effects of axitinib and lenvatinib. Moreover, the results of the *in vivo* experiment further showed that HC-5404 induced greater effects on the axitinib-resistant model^[37]^.

#### Metabolic and angiogenesis pathway inhibitors

Metabolic reprogramming plays a critical role in TKI resistance in RCC, influencing cancer stemness, lipid metabolism, and angiogenesis. Several recent studies have identified metabolic regulators as potential therapeutic targets to enhance sunitinib efficacy. For example, recently, Lv *et al.* developed an engineered CD276-CD133 dual-targeting biomimetic nanovesicle EMφ-siMTHFD2-MnO2@Suni to overcome drug resistance and terminate tumor progression of ccRCC by remodeling folate-nucleotide metabolism^[123]^.

Retinoic acid signaling has been implicated in cancer stemness and acquired resistance to TKIs. Kamada *et al.* (2021) found that dipeptidyl peptidase IV (DPP4) and ALDH1A, key regulators in retinoic acid metabolism, contributed to RCC resistance. Pharmacological inhibition using sitagliptin (DPP4 inhibitor) or disulfiram (ALDH1A inhibitor) effectively restored sunitinib sensitivity, highlighting the therapeutic potential of targeting this pathway^[119]^.

Similarly, SREBP-1-mediated lipid metabolism is another key metabolic driver of resistance. Chen *et al.* (2022) demonstrated that simvastatin, a widely used cholesterol-lowering drug, could inhibit SREBP-1 activity, thereby reversing sunitinib resistance in RCC cells^[120]^. This finding supported the potential repurposing of lipid-lowering agents as anti-cancer adjuvants.

In addition to metabolic rewiring, angiotensin signaling has also been linked to TKI resistance. Khanna *et al.* (2021) reported that angiotensin-(1-7), a heptapeptide generated by ACE2, could synergize with axitinib in VEGFR-resistant ccRCC, offering a novel angiogenesis-modulating strategy to combat resistance^[121]^.

Furthermore, Yin *et al.* (2023) identified a role for HOOK1, a negative regulator of VEGF-1 via TGF-β signaling, in overcoming sunitinib resistance. Their study selected meletin as a HOOK1 agonist, providing a metabolism-related approach to reverse drug resistance through TGF-β pathway modulation^[122]^.

Collectively, these findings underscore the significance of metabolic interventions in overcoming TKI resistance. Targeting retinoic acid metabolism, lipid metabolism, and alternative angiogenic pathways represents a promising strategy to enhance RCC treatment outcomes.

#### Drug repurposing

Drug repurposing, which involves identifying new therapeutic applications for existing FDA-approved drugs, has emerged as a promising strategy to overcome TKI resistance in RCC. Tung *et al.* (2022) identified that penfluridol, a dopamine receptor D2 (DRD2)-targeting antipsychotic, could suppress cancer stemness by inducing autophagy-mediated apoptosis in RCC. Notably, its combination with sunitinib exhibited a synergistic effect, suggesting its potential as an adjunct therapy to enhance TKIs sensitivity^[124]^. Similarly, Greenberg *et al.* (2021) highlighted the role of EVs, particularly exosomes, in promoting TKI resistance. They discovered that ketoconazole (KTZ), an FDA-approved antifungal agent, could inhibit exosome biogenesis and secretion via ERK1/2 suppression, thereby enhancing sunitinib therapeutic effect in resistant 786-O RCC cells^[125]^.

### RNA interference

Non-coding RNAs, including lncRNAs and siRNAs, play crucial roles in the regulation of drug resistance mechanisms in RCC as discussed above. Recent advancements in RNA-based therapeutic strategies have explored innovative nanoparticle delivery systems to counteract sunitinib resistance.

Pan *et al.* investigated the role of IGFL2-AS1, a lncRNA that promoted sunitinib resistance in RCC. To counteract this mechanism, they developed a chitosan-solid lipid nanoparticle system carrying antisense oligonucleotides (ASOs) targeting IGFL2-AS1. This delivery system successfully silenced IGFL2-AS1 expression, effectively restoring sunitinib sensitivity^[45]^.

Similarly, Hua *et al.* developed a Zr-based porphyrinic nanoscale metal-organic framework (PCN-224) for the co-delivery of sunitinib and VEGFR-2-targeting siRNA (siVEGFR-2). To enhance tumor targeting and drug stability, the system was coated with hyaluronic acid (HA), promoting CD44-mediated uptake. This multifunctional platform (St/siVEGFR-2@PCN-224@HA) achieves triple inhibition of tumor growth by integrating targeted therapy, RNA interference, and photodynamic therapy, markedly improving the therapeutic efficacy of TKIs in RCC^[126]^.

These studies highlighted the potential of RNA-based therapeutics in reversing sunitinib resistance. By leveraging lncRNA inhibition (IGFL2-AS1 ASOs) and siRNA-mediated VEGFR-2 suppression through nanoparticle-mediated delivery systems, researchers are developing novel approaches to enhance drug efficacy and tumor selectivity in RCC treatment.

### Others

In 2022, Oosterwijk-Wakka *et al.* reported that SR SK-RC-52 cells expressed pAXL and pMET, unlike the SS NU12 cells. NGS analysis revealed higher expression of VEGFA, VEGFB, VEGFD, PGF, and VEGFR1/2/3 in NU12, while SK-RC-52 exhibited lower VEGFC and PDGFA levels. Notably, combining sunitinib with [177Lu]Lu-cG250 radioimmunotherapy (RIT) achieved the best response in SK-RC-52 tumor-bearing mice after two treatment cycles, suggesting a promising strategy for overcoming sunitinib resistance in RCC^[127]^.

In 2022, Oladejo *et al.* reported that a Listeria-based vaccine encoding an antigenic fragment of CD105 (Lm-LLO-CD105A) showed promising therapeutic efficacy in RCC by targeting both tumor cells and tumor-associated vasculature. This effect was mediated by CD8^+^ T cells and relied on strong CD105 expression in RCC cells^[128]^.

## RECENT CLINICAL TRIALS ADDRESSING TKI RESISTANCE IN RCC (CLINICAL TRIAL LEVEL)

Finally, we reviewed the information on recent clinical trials of combination therapies with TKIs or strategies to overcome TKI resistance in RCC [Table 3]. From Table 3, it is evident that the most common clinical investigations involve the combination of recently FDA-approved HIF-2α inhibitors^[132]^ with TKIs, which aligns with the previously described pivotal role of the HIF-2α pathway in renal cancer angiogenesis, particularly in TKI resistance^[30,49,107,133]^. In addition, combinations with second-line agents such as mTOR inhibitors are also frequently explored.

**Table 3:** Recent clinical trials of combination therapies with TKIs or strategies to overcome TKI resistance in RCC

**NCT number/target**	**Study title**	**Study status**	**Study design**	**Start date**	**Primary completion date**	**Completion Date**	**Locations**
**HIF-2α**							
NCT07097935	Study of HS-10516 Combination Therapy in Patients With Advanced Renal Cell Carcinoma	RECRUITING	Allocation: NA Intervention model: SEQUENTIAL Masking: NONE Primary purpose: TREATMENT	2025/7/10	2027/7/10	2028/7/10	Beijing Cancer Hospital
NCT07011719	Study of Casdatifan and Cabozantinib Versus Placebo and Cabozantinib in Patients With Advanced Clear Cell Renal Cell Carcinoma	NOT_YET_RECRUITING	Allocation: RANDOMIZED Intervention model: PARALLEL Masking: QUADRUPLE (PARTICIPANT, CARE_PROVIDER, INVESTIGATOR, OUTCOMES_ASSESSOR) Primary purpose: TREATMENT	2025/9/1	2028/4/1	2030/12/1	NA
NCT05899049	A Study of Pembrolizumab (MK-3475) in Combination With Belzutifan (MK-6482) and Lenvatinib (MK-7902), or Pembrolizumab/Quavonlimab (MK-1308A) in Combination With Lenvatinib, *vs.* Pembrolizumab and Lenvatinib, for Treatment of Advanced Clear Cell Renal Cell Carcinoma (MK-6482-012)-China Extension Study	ACTIVE_NOT_RECRUITING	Allocation: RANDOMIZED Intervention model: PARALLEL Masking: NONE Primary purpose: TREATMENT	2022/7/27	2026/12/1	2026/12/1	Beijing Cancer Hospital-Renal carcinoma and melanoma (Site 6000)
NCT05536141	A Phase 1 Study of AB521 Monotherapy and Combination Therapies in Renal Cell Carcinoma and Other Solid Tumors	RECRUITING	Allocation: NON_RANDOMIZED Intervention model: SEQUENTIAL Masking: NONE Primary purpose: TREATMENT	2022/10/26	2027/7/1	2027/7/1	University of Alabama at Birmingham
NCT05030506	A Study of Belzutifan (MK-6482) as Monotherapy and in Combination With Lenvatinib (E7080/MK-7902) With or Without Pembrolizumab (MK-3475) in China Participants With Advanced Renal Cell Carcinoma (MK-6482-010)	ACTIVE_NOT_RECRUITING	Allocation: NON_RANDOMIZED Intervention model: PARALLEL Masking: NONE Primary purpose: TREATMENT	2021/10/13	2026/10/21	2026/10/21	Beijing Cancer Hospital-Digestive Oncology (Site 0001)
NCT04736706	A Study of Pembrolizumab (MK-3475) in Combination With Belzutifan (MK-6482) and Lenvatinib (MK-7902), or Pembrolizumab/Quavonlimab (MK-1308A) in Combination With Lenvatinib, Versus Pembrolizumab and Lenvatinib, for Treatment of Advanced Clear Cell Renal Cell Carcinoma (MK-6482-012)	ACTIVE_NOT_RECRUITING	Allocation: RANDOMIZED Intervention model: PARALLEL Masking: NONE Primary purpose: TREATMENT	2021/4/14	2026/10/29	2026/10/29	The University of Alabama at Birmingham (Site 0010)
NCT04626479	Substudy 03A: A Study of Immune and Targeted Combination Therapies in Participants With First Line (1L) Renal Cell Carcinoma (MK-3475-03A)	ACTIVE_NOT_RECRUITING	Allocation: RANDOMIZED Intervention model: PARALLEL Masking: NONE Primary purpose: TREATMENT	2020/12/16	2026/5/31	2026/5/31	University of California at San Francisco (Site 1008)
NCT04586231	A Study of Belzutifan (MK-6482) in Combination With Lenvatinib Versus Cabozantinib for Treatment of Renal Cell Carcinoma (MK-6482-011)	ACTIVE_NOT_RECRUITING	Allocation: RANDOMIZED Intervention model: PARALLEL Masking: NONE Primary purpose: TREATMENT	2021/2/25	2026/2/11	2027/2/11	Ironwood Cancer & Research Centers (Site 0077)
NCT03634540	A Trial of Belzutifan (PT2977, MK-6482) in Combination With Cabozantinib in Patients With Clear Cell Renal Cell Carcinoma (ccRCC) (MK-6482-003)	ACTIVE_NOT_RECRUITING	Allocation: NON_RANDOMIZED Intervention model: PARALLEL Masking: NONE Primary purpose: TREATMENT	2018/9/27	2027/2/26	2027/2/26	USC Norris Comprehensive Cancer Center (Site 0060)
NCT07049926	Substudy 03C: A Study of Combination Therapies in Participants With Renal Cell Carcinoma With Recurrent Disease During or After Anti-PD-(L)1 Therapy (MK-3475-03C/KEYMAKER-U03)	RECRUITING	Allocation: NON_RANDOMIZED Intervention model: PARALLEL Masking: SINGLE (OUTCOMES_ASSESSOR) Primary purpose: TREATMENT	2025/7/20	2031/10/26	2031/10/26	UCSF Medical Center at Mission Bay (Site 5008)
**mTOR**							
NCT05012371	Lenvatinib With Everolimus Versus Cabozantinib for Second-Line or Third-Line Treatment of Metastatic Renal Cell Cancer	ACTIVE_NOT_RECRUITING	Allocation: RANDOMIZED Intervention model: PARALLEL Masking: NONE Primary purpose: TREATMENT	2022/2/16	2025/10/25	2025/10/25	Moffitt Cancer Center
NCT03324373	Lenvatinib and Everolimus in Renal Cell Carcinoma (RCC)	COMPLETED	Allocation: NA Intervention model: SINGLE_GROUP Masking: NONE Primary purpose: TREATMENT	2019/3/20	2023/9/27	2024/8/28	University of Iowa Hospitals and Clinics
NCT03173560	Trial to Assess Safety and Efficacy of Lenvatinib (18 mg *vs.* 14 mg) in Combination With Everolimus in Participants With Renal Cell Carcinoma	COMPLETED	Allocation: RANDOMIZED Intervention model: PARALLEL Masking: NONE Primary purpose: TREATMENT	2017/8/17	2020/2/14	2024/6/20	City of Hope National Medical Center
NCT02915783	A Trial to Evaluate Efficacy and Safety of Lenvatinib in Combination With Everolimus in Subjects With Unresectable Advanced or Metastatic Non Clear Cell Renal Cell Carcinoma (nccRCC) Who Have Not Received Any Chemotherapy for Advanced Disease	COMPLETED	Allocation: NA Intervention model: SINGLE_GROUP Masking: NONE Primary purpose: TREATMENT	2017/2/20	2021/11/2	2021/11/2	H. Lee Moffitt Cancer Center and Research Institute
NCT02811861	Lenvatinib/Everolimus or Lenvatinib/Pembrolizumab Versus Sunitinib Alone as Treatment of Advanced Renal Cell Carcinoma	ACTIVE_NOT_RECRUITING	Allocation: RANDOMIZED Intervention model: PARALLEL Masking: NONE Primary purpose: TREATMENT	2016/10/13	2020/8/28	2026/3/31	Stanford School of Medicine
NCT06317298	Fruquintinib Plus Everolimus as 2nd Line Therapy of ccRCC Patients Progressed Post IO and TKI Therapy	RECRUITING	Allocation: NON_RANDOMIZED Intervention model: SINGLE_GROUP Masking: NONE Primary purpose: TREATMENT	2024/2/21	2025/8/1	2025/12/1	Peking University First Hospital
NCT02479490	Prednisone Plus Everolimus in Patients With Metastatic Renal Cell Cancer After Failure of VEGFR-TKI	TERMINATED	Allocation: NA Intervention model: SINGLE_GROUP Masking: NONE Primary purpose: TREATMENT	2015/9/22	2017/5/5	2017/9/1	U.O Oncologia Medica
**PARP**							
NCT04337970	Talazoparib and Axitinib for People With Previously Treated Advanced Kidney Cancer	COMPLETED	Allocation: RANDOMIZED Intervention model: SEQUENTIAL Masking: NONE Primary purpose: TREATMENT	2020/4/6	2025/5/23	2025/5/23	Memorial Sloan Kettering Basking Ridge (Limited Protocol Activities)
**FTase**							
NCT06026410	KO-2806 Monotherapy and Combination Therapies in Advanced Solid Tumors	RECRUITING	Allocation: NON_RANDOMIZED Intervention model: SEQUENTIAL Masking: NONE Primary purpose: TREATMENT	2023/10/18	2027/1/1	2027/4/1	Mayo Clinic Comprehensive Cancer Center
**HDAC**							
NCT03592472	A Study of Pazopanib With or Without Abexinostat in Patients With Locally Advanced or Metastatic Renal Cell Carcinoma (RENAVIV)	RECRUITING	Allocation: RANDOMIZED Intervention model: CROSSOVER Masking: DOUBLE (PARTICIPANT, INVESTIGATOR) Primary purpose: TREATMENT	2018/7/17	2026/12/30	2028/6/30	University Of UA Cancer Center(UACC)/DH-SJHMC
NCT02795819	Study of the Pan-DAC Inhibitor AR-42 and Pazopanib in Advanced Sarcoma and Kidney Cancer	TERMINATED	Allocation: NON_RANDOMIZED Intervention model: SEQUENTIAL Masking: NONE Primary purpose: TREATMENT	2016/7/8	2016/11/24	2019/3/14	Virginia Commonwealth University/Massey Cancer Center
**Serine/threonine kinase**							
NCT03571438	Evaluation of a Promising New Combination of Protein Kinase Inhibitors on Organotypic Cultures of Human Renal Tumors	UNKNOWN	Allocation: NON_RANDOMIZED Intervention model: PARALLEL Masking: NONE Primary purpose: TREATMENT	2017/10/16	2022/9/30	2024/9/30	Grenoble Alps Hospital
NCT00448721	A Phase II Trial of Perifosine Following Tyrosine Kinase Inhibitor (TKI) - Failure in Patients With Renal Cancer	COMPLETED	Allocation: NA Intervention model: SINGLE_GROUP Masking: NONE Primary purpose: TREATMENT	2007/3/1	2010/10/1	2011/10/1	Investigative Site
**GLS1**							
NCT03428217	CANTATA: CB-839 With Cabozantinib *vs.* Cabozantinib With Placebo in Patients With Metastatic Renal Cell Carcinoma	COMPLETED	Allocation: RANDOMIZED Intervention model: PARALLEL Masking: QUADRUPLE (PARTICIPANT, CARE_PROVIDER, INVESTIGATOR, OUTCOMES_ASSESSOR) Primary purpose: TREATMENT	2018/4/24	2020/8/31	2021/7/16	University of Alabama at Birmingham
NCT02771626	Study CB-839 in Combination With Nivolumab in Patients With Melanoma, Clear Cell Renal Cell Carcinoma (ccRCC) and Non-Small Cell Lung Cancer (NSCLC)	TERMINATED	Allocation: NON_RANDOMIZED Intervention model: FACTORIAL Masking: NONE Primary purpose: TREATMENT	2016/8/1	2020/4/24	2020/4/24	Honor Health
**β-Adrenoceptor blocker**							
NCT03323710	Study of Propranolol Plus Sunitinib in First-line Treatment of Metastatic Renal Cell Carcinoma	WITHDRAWN	Allocation: NA Intervention model: SINGLE_GROUP Masking: NONE Primary purpose: TREATMENT	2025/9/18	2025/12/19	2025/12/19	Military Institute of Medicine
**CXCR4**							
NCT02667886	Trial of X4P-001 in Participants With Advanced Renal Cell Carcinoma	COMPLETED	Allocation: RANDOMIZED Intervention model: PARALLEL Masking: NONE Primary purpose: TREATMENT	2016/4/27	2022/4/14	2022/4/14	Scottsdale
**ENPP3 and tubulin**							
NCT02639182	A Study of AGS-16C3F *vs.* Axitinib in Metastatic Renal Cell Carcinoma	COMPLETED	Allocation: RANDOMIZED Intervention model: PARALLEL Masking: NONE Primary purpose: TREATMENT	2016/5/3	2020/10/2	2020/10/2	Site US01026
**CDK4/6**							
NCT06835972	A Study of Abemaciclib and Cabozantinib in People With Clear Cell Renal Cell Carcinoma (ccRCC)	RECRUITING	Allocation: NA Intervention model: SINGLE_GROUP Masking: NONE Primary purpose: TREATMENT	2025/2/14	2027/2/1	2027/2/1	Johns Hopkins University (Data Collection Only)
NCT05176288	Avelumab, Palbociclib and Axitinib in Advanced RCC	WITHDRAWN	Allocation: NA Intervention model: SINGLE_GROUP Masking: NONE Primary purpose: TREATMENT	2024/5/31	2026/8/31	2027/8/31	
NCT03905889	A Study of Abemaciclib in Combination With Sunitinib in Metastatic Renal Cell Carcinoma	TERMINATED	Allocation: NA Intervention model: SINGLE_GROUP Masking: NONE Primary purpose: TREATMENT	2019/6/5	2022/3/8	2022/3/8	Rhode Island Hospital
**Cell therapy**							
NCT07087158	A Study of IBR854 Combined With Pazopanib Versus Pazopanib in Advanced Renal Cell Carcinoma	NOT_YET_RECRUITING	Allocation: RANDOMIZED Intervention model: PARALLEL Masking: NONE Primary purpose: TREATMENT	2025/8/10	2026/12/31	2026/12/31	The Cancer Hospital of Fudan University
NCT06716853	A Clinical Gene Therapy Study with Hematopoietic Stem Cells for the Treatment, with Single Dose of Temferon, of Patients Suffering from Metastatic Renal Cell Carcinoma	RECRUITING	Allocation: NON_RANDOMIZED Intervention model: PARALLEL Masking: NONE Primary purpose: TREATMENT	2024/10/22	2026/9/30	2026/9/30	Ospedale San Raffaele
NCT05127824	Autologous Dendritic Cell Vaccine in Kidney Cancer	RECRUITING	Allocation: NON_RANDOMIZED Intervention model: SINGLE_GROUP Masking: NONE Primary purpose: TREATMENT	2023/7/6	2026/12/1	2026/12/1	UPMC Department of Urology
NCT04203901	Dendritic Cell Immunotherapy Plus Standard Treatment of Advanced Renal Cell Carcinoma	TERMINATED	Allocation: RANDOMIZED Intervention model: PARALLEL Masking: NONE Primary purpose: TREATMENT	2020/7/22	2023/9/28	2023/9/28	Moffitt Cancer Center
NCT03736330	A Study of Anti-PD-1 Combinations of D-CIK Immunotherapy and Axitinib in Advanced Renal Carcinoma	UNKNOWN	Allocation: NA Intervention model: SINGLE_GROUP Masking: NONE Primary purpose: TREATMENT	2018/9/8	2020/10/8	2021/11/8	Cancer Center
**ADC**							
NCT05620134	Study of JK08 in Patients with Unresectable Locally Advanced or Metastatic Cancer	ACTIVE_NOT_RECRUITING	Allocation: NON_RANDOMIZED Intervention model: SEQUENTIAL Masking: NONE Primary purpose: TREATMENT	2022/10/17	2025/10/17	2026/2/20	Institut Jules Bordet
NCT06962787	A Study of BL-B01D1 + Axitinib Without or With Pembrolizumab in Patients With Locally Advanced or Metastatic Renal Cancer	RECRUITING	Allocation: NA Intervention model: SINGLE_GROUP Masking: NONE Primary purpose: TREATMENT	2025/7/28	2025/5/27	2025/12/27	Fudan University Shanghai Cancer Center
**Radiotherapy**							
NCT02956798	SAbR For Oligometastatic Renal Cell Carcinoma	ACTIVE_NOT_RECRUITING	Allocation: NA Intervention model: SINGLE_GROUP Masking: NONE Primary purpose: TREATMENT	2018/7/19	2025/12/30	2025/12/31	University of Texas Southwestern Medical Center
NCT06889649	SABR Combined with Axitinib and Toripalimab in Recurrent or Metastatic RCC	RECRUITING	Allocation: NA Intervention model: SINGLE_GROUP Masking: NONE Primary purpose: TREATMENT	2019/1/1	2027/2/28	2028/2/20	Peking University First Hospital
NCT06726421	Systemic Therapy Alone or with Stereotactic Body Radiotherapy for Oligometastatic Kidney Cancer (STROKER Study)	RECRUITING	Allocation: RANDOMIZED Intervention model: PARALLEL Masking: SINGLE (OUTCOMES_ASSESSOR) Primary purpose: TREATMENT	2024/9/18	2030/9/30	2033/9/30	Sun Yat-sen University Cancer Center
NCT02307474	A Pilot Study of SBRT With Adjuvant Pazopanib for Renal Cell Cancer	WITHDRAWN	Allocation: NA Intervention model: SINGLE_GROUP Masking: NONE Primary purpose: TREATMENT	2025/9/15	2025/9/15	2025/9/15	Case Medical Center
NCT02599779	A Proof of Principle Study of Pembrolizumab With SBRT in TKI mRCC Patients	COMPLETED	Allocation: NON_RANDOMIZED Intervention model: SINGLE_GROUP Masking: NONE Primary purpose: TREATMENT	2016/12/1	2021/8/1	2021/8/5	Tom Baker Cancer Centre
**Hormone therapy**							
NCT06222593	Study to Evaluate the Safety and Efficacy of Bicalutamide in Combination with Sunitinib in Patients with TKIs-resistant RCC	RECRUITING	Allocation: NA Intervention model: SINGLE_GROUP Masking: NONE Primary purpose: TREATMENT	2024/10/1	2026/7/1	2027/7/1	UB/Great Lakes Cancer Care
NCT03379012	Testosterone in Metastatic Renal Cell Carcinoma Patients	COMPLETED	Allocation: RANDOMIZED Intervention model: PARALLEL Masking: NONE Primary purpose: TREATMENT	2016/2/8	2018/1/30	2019/7/30	A.I. Kryzhanovsky Krasnoyarsk Cancer Center
**Radiation therapy**							
NCT06132945	A Study of Cabozantinib and Nivolumab With Radiation Therapy for People With Renal Cell Carcinoma That Has Spread to the Brain	RECRUITING	Allocation: NA Intervention model: SINGLE_GROUP Masking: NONE Primary purpose: TREATMENT	2023/11/10	2027/11/1	2027/11/1	Memorial Sloan Kettering Basking Ridge (All Protocol Activities)
NCT04071223	Testing the Addition of a New Anti-cancer Drug, Radium-223 Dichloride, to the Usual Treatment (Cabozantinib) for Advanced Renal Cell Cancer That Has Spread to the Bone, RadiCaL Study	RECRUITING	Allocation: RANDOMIZED Intervention model: PARALLEL Masking: NONE Primary purpose: TREATMENT	2020/7/29	2026/10/1	2026/10/1	University of Alabama at Birmingham Cancer Center
NCT02406521	Exploratory Study of Radium-223 and VEGF-Targeted Therapy in Patients With Metastatic Renal Cell Carcinoma and Bone Mets	COMPLETED	Allocation: NON_RANDOMIZED Intervention model: PARALLEL Masking: NONE Primary purpose: TREATMENT	2025/4/15	2025/12/17	2019/12/31	Massachusetts General Hospital
NCT05663710	Phase 1b/2 Study of Combination 177Lu Girentuximab Plus Cabozantinib and Nivolumab in Treatment naïve Patients With Advanced Clear Cell RCC	RECRUITING	Allocation: RANDOMIZED Intervention model: SINGLE_GROUP Masking: NONE Primary purpose: TREATMENT	2023/6/30	2025/10/30	2027/10/30	MD Anderson Cancer Center
**Natural products**							
NCT05363631	Seleno-L Methionine (SLM)-Axitinib-Pembrolizumab	RECRUITING	Allocation: NA Intervention model: SINGLE_GROUP Masking: NONE Primary purpose: TREATMENT	2022/9/19	2026/12/31	2026/12/31	University of Iowa Hospitals & Clinics
NCT02535533	SLM + Axitinib for Clear Cell RCC	COMPLETED	Allocation: NA Intervention model: SINGLE_GROUP Masking: NONE Primary purpose: TREATMENT	2025/1/16	2023/8/22	2025/4/4	University of Iowa Hospitals and Clinics
NCT05122546	CBM588 in Combination With Nivolumab and Cabozantinib for the Treatment of Advanced or Metastatic Kidney Cancer	ACTIVE_NOT_RECRUITING	Allocation: RANDOMIZED Intervention model: PARALLEL Masking: NONE Primary purpose: TREATMENT	2021/11/1	2025/10/25	2025/10/25	City of Hope Medical Center
NCT03334409	Pazopanib Hydrochloride With or Without Ascorbic Acid in Treating Patients With Kidney Cancer That Is Metastatic or Cannot Be Removed by Surgery	TERMINATED	Allocation: RANDOMIZED Intervention model: PARALLEL Masking: NONE Primary purpose: TREATMENT	2018/2/16	2021/3/13	2021/3/13	Illinois CancerCare-Peoria
NCT02446795	Isoquercetin as an Adjunct Therapy in Patients With Kidney Cancer Receiving First-line Sunitinib: a Phase I/II Trial	UNKNOWN	Allocation: RANDOMIZED Intervention model: PARALLEL Masking: TRIPLE (PARTICIPANT, CARE_PROVIDER, INVESTIGATOR) Primary purpose: TREATMENT	2025/11/16	2025/12/17	2025/12/17	Azienda Ospedaliera Cardarelli Divisione Di Oncologia
**Switch TKIs or with a higher dose**							
NCT05931393	Sequential Treatment of Cabozantinib for Advanced Renal Cell Carcinoma (RCC)	RECRUITING	Allocation: NON_RANDOMIZED Intervention model: SEQUENTIAL Masking: NONE Primary purpose: TREATMENT	2023/12/20	2025/9/1	2027/12/31	UT Southwestern Medical Center
NCT05678673	Study of XL092 + Nivolumab *vs.* Sunitinib in Subjects With Advanced or Metastatic Non-Clear Cell Renal Cell Carcinoma	ACTIVE_NOT_RECRUITING	Allocation: RANDOMIZED Intervention model: PARALLEL Masking: NONE Primary purpose: TREATMENT	2023/1/1	2025/7/25	2025/6/28	Exelixis Clinical Site #1
NCT05522231	Efficacy and Safety of Fruquintinib in Combination With Sintilimab in Advanced Renal Cell Carcinoma (FRUSICA-2)	ACTIVE_NOT_RECRUITING	Allocation: RANDOMIZED Intervention model: PARALLEL Masking: NONE Primary purpose: TREATMENT	2022/10/27	2025/1/25	2025/3/25	Beijing Chao-Yang Hospital
NCT04609800	Study on the Utilization of Cabozantinib in Adult Patients With Advanced or Metastatic Renal Cell Carcinoma (RCC) in 2nd Line Treatment Following Prior Vascular Endothelial Growth Factor (VEGF)-Targeted Therapy Under Real-real Life Clinical Setting in France	WITHDRAWN	Observational model: Cohort Time perspective: Prospective	2020/11/6	2023/5/10	2023/5/10	
NCT04458259	Study of PF-07265807 in Participants With Metastatic Solid Tumors	TERMINATED	Allocation: NON_RANDOMIZED Intervention model: SEQUENTIAL Masking: NONE Primary purpose: TREATMENT	2020/9/24	2024/11/15	2024/11/15	Henry Eye Clinic
NCT04300140	Safety and Efficacy Study of AVB-S6-500 (Batiraxcept) in Patients With Advanced or Metastatic Clear Cell Renal Cell Carcinoma	TERMINATED	Allocation: RANDOMIZED Intervention model: SINGLE_GROUP Masking: NONE Primary purpose: TREATMENT	2021/2/26	2023/8/14	2023/8/14	University of Maryland Greenebaum Comprehensive Cancer Center
NCT02867592	Cabozantinib-S-Malate in Treating Younger Patients With Recurrent, Refractory, or Newly Diagnosed Sarcomas, Wilms Tumor, or Other Rare Tumors	ACTIVE_NOT_RECRUITING	Allocation: NA Intervention model: SINGLE_GROUP Masking: NONE Primary purpose: TREATMENT	2017/5/18	2021/6/30	2026/6/27	Children’s Hospital of Alabama
NCT02761057	Testing Cabozantinib, Crizotinib, Savolitinib and Sunitinib in Kidney Cancer Which Has Progressed	COMPLETED	Allocation: RANDOMIZED Intervention model: PARALLEL Masking: NONE Primary purpose: TREATMENT	2016/7/25	2023/10/19	2023/10/19	Anchorage Associates in Radiation Medicine
NCT02122003	Second Line Sorafenib After Pazopanib in Patients With RCC	TERMINATED	Allocation: NA Intervention model: SINGLE_GROUP Masking: NONE Primary purpose: TREATMENT	2025/9/16	2017/11/8	2017/11/8	Istituto Tumori
**Oncologic physical therapy**							
NCT05092373	Phase I Study of Tumor Treating Fields (TTF) in Combination With Cabozantinib or With Pembrolizumab and Nab-Paclitaxel in Patients With Advanced Solid Tumors Involving the Abdomen or Thorax	RECRUITING	Allocation: NON_RANDOMIZED Intervention model: PARALLEL Masking: NONE Primary purpose: TREATMENT	2022/4/29	2026/9/1	2026/9/1	M D Anderson Cancer Center
**Immune related**							
**CD80**							
NCT04977453	GI-101 as a Single Agent or in Combination With Pembrolizumab, Lenvatinib or Local Radiotherapy in Advanced Solid Tumors	RECRUITING	Allocation: NON_RANDOMIZED Intervention model: SEQUENTIAL Masking: NONE Primary purpose: TREATMENT	2021/8/2	2025/10/26	2025/12/26	Tisch Cancer Institute (TCI)
**IL2**							
NCT04540705	A Study to Compare Bempegaldesleukin (BEMPEG: NKTR-214) Combined With Nivolumab and Tyrosine Kinase Inhibitor (TKI) to Nivolumab and TKI Alone in Participants With Previously Untreated Kidney Cancer That is Advanced or Has Spread	COMPLETED	Allocation: NON_RANDOMIZED Intervention model: PARALLEL Masking: NONE Primary purpose: TREATMENT	2020/9/11	2024/1/18	2024/1/18	Local Institution - 0005
**LILRB2**							
NCT04626518	Substudy 03B: A Study of Immune and Targeted Combination Therapies in Participants With Second Line Plus (2L+) Renal Cell Carcinoma (MK-3475-03B/KEYMAKER-U03)	ACTIVE_NOT_RECRUITING	Allocation: RANDOMIZED Intervention model: PARALLEL Masking: NONE Primary purpose: TREATMENT	2020/12/17	2026/5/31	2026/5/31	University of California at San Francisco (Site 3008)
**IL-1β**							
NCT03798626	Gevokizumab With Standard of Care Anti-cancer Therapies for Metastatic Colorectal, Gastroesophageal, and Renal Cancers	COMPLETED	Allocation: NON_RANDOMIZED Intervention model: PARALLEL Masking: NONE Primary purpose: TREATMENT	2019/5/22	2023/3/1	2025/2/5	University of California LA
**OX40**							
NCT03092856	Axitinib With or Without Anti-OX40 Antibody PF-04518600 in Treating Patients With Metastatic Kidney Cancer	ACTIVE_NOT_RECRUITING	Allocation: RANDOMIZED Intervention model: PARALLEL Masking: TRIPLE (PARTICIPANT, CARE_PROVIDER, INVESTIGATOR) Primary purpose: TREATMENT	2017/7/19	2025/12/31	2026/12/31	Los Angeles County-USC Medical Center
**PTPN2/PTPN1**							
NCT04777994	Study With ABBV-CLS-484 in Participants With Locally Advanced or Metastatic Tumors	RECRUITING	Allocation: NON_RANDOMIZED Intervention model: SEQUENTIAL Masking: NONE Primary purpose: TREATMENT	2021/3/9	2026/10/1	2026/10/1	University of Arizona Cancer Center - Tucson / ID# 262698
**Dietary intervention**							
NCT02729194	Pilot Study of Pazopanib With Low Fat Meal (PALM) in Advanced Renal Cell Carcinoma	COMPLETED	Allocation: NA Intervention model: SINGLE_GROUP Masking: NONE Primary purpose: TREATMENT	2025/6/16	2025/9/17	2025/9/17	University of Michigan Comprehensive Cancer Center

TKIs: Tyrosine kinase inhibitors; RCC: renal cell carcinoma.

Another interesting trend is that researchers are beginning to explore the combination of TKIs with immunomodulatory agents beyond the conventional first-line ICIs (PD-1/PD-L1/CTLA-4), aiming for enhanced efficacy. Even various cell therapies are now being tested in combination with TKIs, providing potential guidance for the future direction of pharmacological treatment in renal cancer.

Notably, some natural agents, including vitamin C and the gut microbiota modulator CBM588, are FDA-approved nutraceuticals or probiotics. This regulatory status suggests that such combination strategies may be more readily translatable into clinical application once supportive preclinical or early clinical evidence is obtained^[134,135]^.

However, some clinical trials are still primarily focused on either using the next-generation TKIs or simply increasing drug dosages. However, based on previous experience, the pace of drug development and clinical testing for agents with similar mechanisms generally may not keep pace with the rate of resistance development in patients. Therefore, combination strategies involving drugs with distinct mechanisms should be the primary focus of future investigations.

## DISCUSSION AND CONCLUSION

### The role of TKIs in RCC and the challenge of resistance

TKIs have been a cornerstone in the treatment of RCC, particularly for advanced cases. These drugs primarily target tumor angiogenesis by inhibiting VEGFR, PDGFR, and other pathways that drive neovascularization. However, despite their clinical efficacy, the emergence of resistance remains an inevitable challenge. Over time, RCC tumors adapt to TKI therapy through a variety of mechanisms, rendering these treatments less effective and leading to disease progression^[2,4,12,136]^.

### Current landscape of TKI resistance mechanisms in RCC

A review of studies from 2020 to 2025 indicates that research on TKI resistance in RCC has largely focused on bypass pathway activation^[32,33]^, the roles of lncRNAs^[45,47]^ and circRNAs^[41,42]^, and forms of regulated cell death, such as apoptosis^[108]^ and ferroptosis^[53]^. However, a crucial aspect of RCC biology - metabolic reprogramming^[137]^ - remains relatively underexplored in the context of TKI resistance. Given that metabolic adaptations play a significant role in RCC progression and therapy resistance, more research is needed to uncover metabolic vulnerabilities that could be exploited to overcome TKI resistance.

Additionally, while significant efforts have been made to investigate resistance mechanisms across various TKIs, most studies still primarily focus on sunitinib. This focus may be influenced by the increasing use of ICIs and other novel therapies, which have shifted research priorities. However, as TKIs remain a cornerstone of RCC treatment, resistance studies should extend to newer-generation TKIs and explore common resistance mechanisms across different TKIs. Furthermore, current strategies to overcome resistance mostly involve TKI combination therapies; nevertheless, systematic comparisons of their efficacy and toxicity have been limited. Future research should prioritize optimizing these combination strategies while ensuring their safety and clinical applicability.

### Limitations of current research and the need for large-scale studies

While significant progress has been made in understanding traditional resistance mechanisms, most studies have remained limited in scale and scope. Many investigations focused on a single drug within a single RCC model, making it challenging to generalize findings across different tumor subtypes or treatment regimens. There is an urgent need for large-scale, multi-model studies conducted by research groups with the necessary resources to systematically examine TKI resistance across various settings. A notable example of such an approach is the highly systematic study by Zhang *et al.* in 2023, which closely reflects real-world clinical scenarios^[138]^. Expanding such efforts would provide invaluable foundational data to guide future research, particularly for groups with more constrained experimental capabilities. At present, studies on TKI resistance in RCC remain relatively isolated, with investigations of the immune microenvironment largely focused on mechanisms of ICI responsiveness while neglecting its interplay with TKI therapy. In real-world clinical settings, however, acquired resistance arising from combined TKI and ICI treatment should be regarded as an integrated and complex process rather than two independent phenomena. Future exploration of resistance mechanisms - whether to TKIs alone or to TKI–ICI combinations - should place greater emphasis on the intricate regulatory role of the immune microenvironment.

### Unique aspects of TKI resistance in RCC: tumor-angiogenesis dependency

Unlike TKIs in cancers such as lung cancer, which target oncogenic driver mutations (e.g., EGFR, ALK, ROS1 alterations)^[139]^, TKIs in RCC predominantly function by disrupting tumor angiogenesis rather than directly targeting tumor cells. ccRCC, the most common subtype, is characterized by frequent inactivation of the VHL tumor suppressor gene, leading to constitutive stabilization of HIFs and subsequent upregulation of VEGF and PDGF^[10,11]^. Consequently, RCC tumors exhibit strong dependence on neovascularization for sustained growth and survival. TKIs such as sunitinib, pazopanib, cabozantinib, and lenvatinib exert their effects by targeting VEGFR, PDGFR, and c-KIT - receptors primarily expressed on endothelial cells rather than the tumor cells themselves.

This fundamental difference in mechanism alters the patterns of resistance observed in RCC. While many cancers develop resistance through secondary kinase domain mutations^[140-142]^, RCC tumors more commonly adapt by modifying their TME, increasing HIF-2α signaling, and undergoing metabolic reprogramming. Unfortunately, most studies on RCC TKI resistance focus on direct tumor cell responses, neglecting the role of endothelial cells and the broader TME. This presents a major gap in current research: instead of solely examining the direct effects of TKIs direct effects on RCC cells, it may be more insightful to investigate how RCC tumors adapt to vascular disruption and reestablish tumor progression. Just as RCC-immune cell interactions have been extensively studied in the context of immunotherapy resistance, there is a pressing need to explore RCC-endothelial cell interactions in the context of TKI resistance.

### Future directions: leveraging advanced technologies to address knowledge gaps

Addressing the complexities discussed above requires advancements in both methodology and model systems. While technological and experimental limitations have constrained past research, emerging techniques such as single-cell sequencing, spatial transcriptomics and metabolomics, hemodynamic and vascularized organoids modeling offer promising new avenues^[143-146]^. These technologies enable deeper insights into the interplay between TKIs, RCC cells, and the surrounding endothelial network. Future studies should leverage these tools to explore resistance mechanisms at a higher resolution, ultimately guiding the development of more effective therapeutic strategies that consider both tumor-intrinsic and microenvironmental adaptations.

In summary, while significant progress has been made in understanding RCC TKI resistance, critical gaps remain. Future research should expand beyond sunitinib to newer TKIs, explore metabolic reprogramming as a key resistance mechanism, and incorporate large-scale, systematic studies. Shifting focus from tumor-centric to microenvironment-centric approaches may provide novel insights into overcoming resistance. With ongoing advancements in technology and research methodologies, future studies have the potential to refine therapeutic strategies and improve patient outcomes in RCC.

## DECLARATIONS

### Copyright

© The Author(s) 2025.
